# Age-dependent changes of the antioxidant system in rat livers are accompanied by altered MAPK activation and a decline in motor signaling

**DOI:** 10.17179/excli2015-734

**Published:** 2015-12-22

**Authors:** Wei Yang, Britta Burkhardt, Luise Fischer, Maja Beirow, Nadja Bork, Eva C. Wönne, Cornelia Wagner, Bettina Husen, Katrin Zeilinger, Liegang Liu, Andreas K. Nussler

**Affiliations:** 1 Eberhard Karls University Tübingen, Dept. of Traumatology, Schnarrenbergstr. 95, 72076 Tübingen, Germany; 2 Department of Nutrition and Food Hygiene, School of Public Health, Tongji Medical College, Huazhong University of Science and Technology, 13 Hangkong Road, Wuhan 430030, PR China; 3 Berlin-Brandenburg Center for Regenerative Therapies (BCRT), Charité Universitätsmedizin Berlin, Campus-Virchow-Klinikum, Augustenburger Platz 1, 13353 Berlin, Germany; 4 Pharmacelsus GmbH, Science Park 2, 66123 Saarbrücken, Germany

**Keywords:** aging, liver, antioxidant, Akt, MAPKs, cell proliferation

## Abstract

Aging is characterized by a progressive decrease of cellular functions, because cells gradually lose their capacity to respond to injury. Increased oxidative stress is considered to be one of the major contributors to age-related changes in all organs including the liver. Our study has focused on elucidating whether important antioxidative enzymes, the mTOR pathway, and MAPKs exhibit age-dependent changes in the liver of rats during aging. We found an age-dependent increase of GSH in the cytosol and mitochondria. The aged liver showed an increased SOD enzyme activity, while the CAT enzyme activity decreased. HO-1 and NOS-2 gene expression was lower in adult rats, but up-regulated in aged rats. Western blot analysis revealed that SOD1, SOD2, GPx, GR, γ-GCL, and GSS were age-dependent up-regulated, while CAT remained constant. We also demonstrated that the phosphorylation of Akt, JNK, p38, and TSC2^Ser1254^ decreased while ERK1/2 and TSC2^Thr1462^ increased age-dependently. Furthermore, our data show that the mTOR pathway seems to be activated in livers of aged rats, and hence stimulating cell proliferation/regeneration, as confirmed by an age-dependent increase of PCNA and p-eIF4E^Ser209 ^protein expression. Our data may help to explain the fact that liver cells only proliferate in cases of necessity, like injury and damage. In summary, we have demonstrated that, age-dependent changes of the antioxidant system and stress-related signaling pathways occur in the livers of rats, which may help to better understand organ aging.

## Abbreviations

1,1,3,3-Tetraethoxypropane, TEP. 4-hydroxy nonenal, 4-HNE. Alanine transaminase, ALT. Bovine serum albumin, BSA. Catalase, CAT. C-Jun N-terminal kinases, JNK. Eukaryotic translation initiation factor 4E, eIF4E. Extracellular signal-regulated kinases, ERK. Eukaryotic translation initiation factor 4E-binding protein 1, 4E-BP1. Glutathione reductase, GR. Glutathione peroxidase, GPx. Glutathione, GSH. Glutathione synthetase, GSS. Lactate dehydrogenase, LDH. γ-glutamylcysteine-ligase, γ-GCL. Mammalian target of rapamycin, mTOR. Mitogen-activated protein kinases, MAPKs. Malondialdehyde, MDA. Nitric oxide synthase, NOS. Proliferating cell nuclear antigen, PCNA. Reactive oxygen species, ROS. Superoxide dismutase 1 and 2, SOD1 and SOD2. Thiobarbituric acid, TBA. Tuberous sclerosis complex 2, TSC2.

## Introduction

According to the World Health Organization (WHO), the proportion of the world population which is aged over 60 years will double from about 11 to 22 % between 2000 and 2050 [http://www.who.int/ageing/en/]. An investigation of the leading causes of death in the USA (International Reference Life Cycle Data System, ILCD) and in the EU has demonstrated that, each year, approximately 100,000 people die from chronic liver diseases [http://www.elpa-info.org]. Besides alcohol, virus infections and accidental injuries, aging is one of the important factors causing the development of chronic liver disease (Junaidi and Di Bisceglie, 2007[17]; Grizzi et al., 2013[9]). Deaths due to liver diseases are 3 to 5-fold higher in people aged over 65 years than in those under 45 years of age (Regev and Schiff, 2001[39]).

Growing old is a normal event in the life of virtually all organisms (Harman, 1956[10]). Aging is characterized by a progressive decrease of cellular functions, because cells progressively lose their capacity to respond to injury (Cui et al., 2012[6]). It is believed that this loss is triggered by environmental influences, such as diet, lack of exercise as well as the exposure to toxic chemicals (Migliore and Coppede, 2009[28]). In comparison to other organs, age-related changes of the liver have been considered to be of minor importance so far (Junaidi and Di Bisceglie, 2007[17]). It is well-known that, with increasing age, the liver undergoes atrophy, that its weight is reduced by about 25-35 %, and that its blood flow decreases (Zeeh and Platt, 2002[56]). The brown atrophy is due to an accumulation of highly oxidized insoluble proteins (lipofuscin) in hepatocytes. It is supposed that lipofuscin is related to chronic oxidative stress and a failure to degrade damaged and denatured proteins (Grizzi et al., 2013[9]). Therefore, oxidative stress is considered to be one of the major contributors to age-related changes in mammals including changes in the liver. It has been suggested that, during aging, the increase in oxidative stress and the subsequent molecular damage are the result of an augmented formation of reactive oxygen species (ROS) and of a greater susceptibility of tissues to oxidative injury, which leads to various diseases (Harman, 1956[10]; Kitani, 2007[19]; Lee et al., 2004[23]; Migliore and Coppede, 2009[28]; Grizzi et al., 2013[9]). These effects are, at least in part, caused by alterations in the enzyme activities and substrate concentrations, especially in the antioxidant system (Szaleczky et al., 1999[46]). The liver contains a battery of enzymes with antioxidative functions, like *e.g.* cytosolic superoxide dismutase (SOD1, Cu/Zn-SOD), mitochondrial SOD (SOD2, Mn-SOD), as well as peroxisomal catalase (CAT) (Barja de Quiroga et al., 1990[2]; Weydert and Cullen, 2010[52]). Furthermore, enzymes associated with glutathione (GSH) synthesis and transformation, such as glutathione peroxidase (GPx) and glutathione reductase (GR), are directly or indirectly involved in the detoxification of ROS. Furthermore, these enzymes are responsible for the GSH homeostasis (Zhu et al., 2006[60]). GSH is the most important soluble antioxidant. Moreover, it reaches cytosolic concentrations of about 10-15 mM (Mari et al., 2009[25]). Altered activities of antioxidant enzymes as well as the impairment of GSH recycling result in an increased cellular accumulation of ROS which damages cellular macromolecules and leads to dysfunctions of organelles, such as the mitochondria (Cui et al., 2012[6]).

Throughout evolution, most organisms have developed mechanisms that enable them to change efficiently between anabolic and catabolic states. These mechanisms allow them to survive and grow in environments with different availabilities of nutrients. An example of such a mechanism in mammals is the signaling network that is anchored to the mammalian target of rapamycin (mTOR), and that responds to diverse environmental cues and controls many processes that produce or use large amounts of energy, nutrients or growth factors, such as cell growth, proliferation, and survival (Laplante and Sabatini, 2012[21]). The direct relationship between mTOR signaling and longevity has been demonstrated for the first time in *S*. *cerevisiae*, *C. elegans* and *D. melanogaster* (Katewa and Kapahi, 2011[18]). After the treatment with the mTOR inhibitor rapamycin, an increased life span has been reported in mice (Neff et al., 2013[30]).

However, there exist contradicting descriptions of the interaction between mitogen-activated protein kinases (MAPK) and the mTOR pathways in different tissues during aging: For example, Hernández et al. (2011[11]) have reported the existence of a protective pathway in cardiomyocytes which involves p38 and Akt-mediated mTOR activation in an ischemia/reperfusion model of C75/B16 mice, while other researchers have postulated an increased phosphorylation of MAPK (e.g. p38) and mTOR in branchial arch muscles from 8- to 26-months-old F344 rats (Bodine et al., 2001[3]). Other scientists have reported a declining phosphorylation of ERK and p70S6 kinase (p70S6K) Thr421/Ser424 with increasing age in the biceps brachii. This finding suggests that the phosphorylation of Akt and MAPK activates mTOR in order to increase the proliferation of muscle satellite cells (Rahnert et al., 2011[37]; Bodine et al., 2001[3], Anjum and Blenis, 2008[1]). Previous publications have suggested the existence of the ROS-induced activation of MAPK pathways and age-dependent changes in the activation status of MAPK in various tissues, including brain, lung, muscle, and liver (Son et al., 2011[45]). It has been demonstrated that, in a multicellular organism, the expression of p38 declines with increasing age (Hsieh et al., 2003[13]). Furthermore, the p-ERK1/2 has been down-regulated in the brain of 24-month-old Fischer 344 rats (Zhen et al., 1999[59]; Youngman et al., 2011[55]). In contrast, an increase in p38 phosphorylation has been observed in the lung and brain of mice, while it has not been detected in the liver (Li et al., 2011[24]). Furthermore, an activation of JNK and p38 signaling has been reported in the livers of aged male mice (Hsieh and Papaconstantinou, 2002[12]; Hsieh et al., 2003[13]). So far, little is known about the effects of oxidative stress on the expression of the Akt-mTOR-p70S6K and MAPKs-mTOR-p70S6K pathways during the aging process of the liver.

Therefore, our aim consists in elucidating whether there exist age-dependent changes of the mTOR pathway and of related MAPKs in the liver of Wistar rats. Furthermore, we link these findings to the (anti-) oxidant status of the livers of male Wistar rats of different ages.

## Materials and Methods

### Materials

All chemicals and reagents have been obtained from Sigma Aldrich (Taufkirchen, Germany), Carl Roth (Karlsruhe, Germany) or PAA Laboratories (Cölbe, Germany). Primers for RT-PCR have been acquired from Eurofins MWG Operon (Ebersberg, Germany). The monoclonal antibodies (CAT, GR, GPx, SOD1, SOD2, GSS, phospho-p38^Thr180/Tyr182^, phospho-JNK^Thr183/Tyr185^, phospho-ERK_1/2_^Thr202/Thr204^, phospho-Akt^Ser473^, phospho-mTOR^Ser2448^, phospho-TSC2^Thr1462^, phospho-TSC2^Ser1254^, phospho-p70s6k^Thr389^, PCNA, nitrotyrosine and *β*-actin), as well as the polyclonal antibodies (GCLC, phospho-eIF4E^Ser209^, and GAPDH) have been purchased from Cell Signaling Technology (Danvers, MA, USA), Santa Cruz, (Dallas, TX, USA), Abcam, (Cambridge, MA, USA), Thermo Fisher Scientific (Waltham, MA, USA), Sigma Aldrich (St. Louis, MO, USA), and Millipore Corporation (Billerica, MA, USA).

### Animals

Wistar rats (RjHan:WI) of different ages were purchased from Janvier Labs (Genest-St-Isle, France), housed in a temperature-controlled room (20-24 °C) and maintained in a 12 h light/12 h dark cycle. Young (7 weeks, N = 8), adult (8 months, N = 10), and aged (23 months, N = 9) rats were included in this study. Sample collection was started after an acclimatization period of at least 1 week. All experimental procedures were approved by and conducted in accordance with the regulations of the local Animal Welfare authorities (Landesamt für Gesundheit und Verbraucherschutz, Abteilung Lebensmittel- und Veterinärwesen, Saarbrücken, file number C1 2.4.2.2 Nr.12/2012). The rats were subjected to fasting overnight. Rats were sacrificed and blood was collected under isoflurane anesthesia by cardiac puncture for exsanguination and the preparation of serum on the next day. Finally, the livers were dissected for the preparation of tissue samples. 

### Preparation of rat liver cytosolic and mitochondria-enriched fractions

The isolation of cytosol and mitochondria from rat liver tissue was performed according to a protocol from Lake (Lake, 1987[20]): 500 mg tissue were homogenized in 500 µl of homogenization buffer (25 mM HEPES, 1.5 mM EDTA, 0.1 M NaCl, 1 % Glycine in ddH_2_O) with ceramic beads (Precellys Ceramic Kit 1.4/2.8 mm, Peqlab, Erlangen, Germany) in a Disruptor Genie (Scientific Industries, Bohemia, NY, USA) at 2,700 rpm for 1 min (~10 disruption cycles; samples were chilled on ice in-between). Cell nuclei and tissue debris were eliminated by centrifugation (800 g, 4 °C, 30 min); supernatants were transferred into pre-cooled Eppendorf tubes and centrifuged again (6,000 g, 4 °C, 15 min). Then, these supernatants (cytosolic fraction) were transferred to new Eppendorf tubes, and the cell pellets (mitochondria-enriched fraction) were washed once again and finally re-suspended in 100 μl resuspension buffer (0.15 M KCl, 50 mM Tris, 1 mM EDTA in ddH_2_O). The measurement of lactate dehydrogenase (LDH) activity was used to ensure that the mitochondria-enriched fraction is cytosol-free. The protein contents were measured by Micro Lowry assay.

### Measurement of GSH and GSSG in serum, liver cytosolic, and mitochondria-enriched fractions

The GSH recycling assay was performed according to a protocol by Rahman et al. (2006[36]): The protein precipitation of subcellular fractions was carried out with 5 % m-phosphoric acid. After 10 min of incubation at 4 °C, the samples were centrifuged at 12,000 g and 4 °C for 10 min. Besides, serum was deproteinized with Roti^®^-spin MINI-10 according to the manufacturer's instructions (Carl Roth GmbH, Karlsruhe, Germany). After the re-neutralization of subcellular fractions [5 mM EDTA solute in 0.1 M potassium phosphate buffer (pH = 7.4)], total GSH were determined as follows: Solutions of 5,5'-dithiobis-2-nitrobenzoic acid (DTNB, 1.68 mM), NADPH (0.8 mM) and GR (2.4 U/ml) were prepared in 0.1 M potassium phosphate buffer (pH = 7.4) containing 5 mM EDTA. The GSH standards were prepared between 0.103 and 26.4 nmol/ml. For the determination of total GSH, 20 µl of standards or samples were incubated with 120 µl of a DTNB/GR mixture (1:1) for 30 seconds. Then, 60 µl of NADPH were added and absorbance was measured at 412 nm for 10 minutes. In order to determine GSSG, free GSH was masked with 2-vinylpyridine. Therefore, m-phosphoric acid had to be added to the deproteinized serum. These samples of serum and the supernatants of cytosolic and mitochondria-enriched fractions were incubated with 3.7 % vinylpyridine for 1 h at room temperature. After re-neutralization with 0.1 M K_2_HPO_4_ (pH = 8.0) [containing 5 mM EDTA], the recycling assay was performed as described above. Total and oxidized glutathione were calculated with the linear regression of the GSH standard curve and were expressed as nmol/mg protein.

### Measurement of enzyme activities in the cytosolic fractions of rat liver

The measurements of the enzyme activity have been carried out as described below by using a FLUOstar Omega Fluorometer (BMG Labtech, Offenburg, Germany).

### CAT and SOD measurement

The CAT activity in liver cytosolic fractions was measured with the fluorometric catalase activity kit (Cell Biolabs, San Diego, CA, USA) according to the manufacturer's instructions. Fluorescence was measured at 544 nm (λ_ex_) and 590 nm (λ_em_). Catalase activity (U/ml) was calculated from a standard curve by using a second order polynomial trendline (y = ax^2^ + bx + c) and expressed as µmol/min/mg protein. In order to measure the liver cytosolic SOD activity, a commercially available kit (Sigma Aldrich, Taufkirchen, Germany) was used according to the manufacturer's protocol. Absorbance was measured at 450 nm every 5 min over 1 h. ΔE in the linear range was used for further calculations. SOD activity in U/ml was calculated by linear regression of the SOD standard curve and expressed as µmol/min/ mg protein.

### LDH, ALT, and 4-HNE

LDH and ALT activity were measured in rat serum with commercially available reaction kits according to the manufacturer's instructions (Analyticon^®^ Biotechnologies AG, Lichtenfels, Germany). Absorbance was measured at 340 nm for 3 min. The calculation of ALT and LDH activities (U/ml) was carried out according to the manufacturer's protocol. The 4-HNE in serum samples have been detected by ELISA kit (CUSABIO, Wuhan, China). The 4-HNE serum levels have been expressed as ng/ml.

### Glutathione Peroxidase (GPx)

The measurement of GPx activity in liver cytosolic fractions was performed according to a previously published protocol (Weydert and Cullen, 2010[52]). Cumene hydroperoxide was used as a substrate for GPx. A GPx standard curve was prepared between 0.1 and 1 U/ml in 0.05 M potassium phosphate buffer (pH = 7.0). A GPx assay solution (1.33 U/ml GR, 1.33 mM GSH in 0.05 mM potassium phosphate buffer (pH = 7.0) containing 1.1 mM EDTA and 1.1 mM NaN_3_) was prepared. In addition, solutions of 15 mM cumene hydroperoxide in ddH_2_O and 4 mM NADPH in 0.05 M potassium phosphate buffer (pH = 7.0) were prepared. 10 μl of each GPx standard or sample were mixed with 15 μl of NADPH, and 75 μl of GPx assay solution and incubated at RT for 5 minutes. Then, 10 μl of cumene hydroperoxide solution were added and the decrease of absorbance at 340 nm was measured during 15 min. ΔE in the linear range was used for further calculations. Linear regression of the GPx standard curve was performed, and values were expressed as µmol/min/mg protein.

### Glutathione Reductase (GR)

The GR activity assay has been performed as described by Smith et al. (1988[44]). A reaction mixture containing DTNB (0.8 mM), NADPH (0.1 mM), and EDTA (1 M) in 0.2 M potassium phosphate buffer (pH = 7.5) as well as a GSSG working solution (20 mM) in 0.2 M potassium phosphate buffer (pH = 7.5) were prepared. For the standard curve, GR was diluted to the final concentrations of 0.1-0.8 U/ml in potassium phosphate buffer (0.2 M, pH = 7.5). 5 μl of each GR standard or sample were mixed with 185 μl reaction mixture and 10 μl GSSG solution. Then, the increase in absorbance at 412 nm was measured during 15 min. ΔE in the linear range was used for further calculations. Linear regression of the GR standard curve was performed, and values were expressed as µmol/min/mg protein.

### RT-PCR for gene expression of antioxidant enzymes 

Conventional reverse transcription-polymerase chain reaction analysis was carried out according to a previously established protocol (Schyschka et al., 2013[43]). First, RNA was extracted by the Trifast reagent according to the manufacturer's guidelines (Peqlab, Erlangen, Germany). The cDNA synthesis was performed using the First Strand cDNA Synthesis kit (Thermo Scientific, Sankt Leon-Rot, Germany). Primer sequences were designed using the primer blast program and synthesized by MWG Operon (Eurofins, Ebersberg, Germany). The applied primers are listed in Table 1(Tab. 1). The appropriate cDNA dilution and PCR cycling numbers for each gene were determined in order to ensure that the PCRs did not reach saturation. PCR was performed using the KAPA2G Fast ReadyMix with dye (peqlab Biotechnologie GmbH, Erlangen, Germany) according to the manufacturer's guidelines. Denaturation was performed at 95 °C for 15 seconds, then the primers were annealed at the appropriate temperatures (Table 1(Tab. 1)) for 15 seconds and finally the elongation was carried out at 72 °C for 10 seconds. The products were resolved by gel electrophoresis in a 1.5 % (w/v) agarose gel in Tris-borate EDTA, and visualized with ethidium bromide. Densitometric analysis was carried out with the ImageJ software (National Institutes of Health, USA).

### Protein extraction and Western Blot analysis

The extraction of tissue protein was performed according to a previously established protocol (Ehnert et al., 2012[7]). Rat liver tissue (30 mg) was suspended in 100 μl ice-cold radioimmunoprecipitation assay (RIPA) lysis buffer (1.5 mM MgCl_2_, 10 mM KCl, 1 mM dithiothreitol, 10 mM Hepes, pH = 7.9) containing a mix of protease and phosphatase inhibitors according to the manufacturer's instructions. Ceramic beads were used for homogenization following the manufacturer's instruction manual. After 10 min of centrifugation at 10,000 g (4 °C), aliquots of supernatants (50 µg protein) were separated by 6 %, 10 % or 12 % SDS-polyacrylamide gel and then transferred onto a nitrocellulose membrane (Carl Roth, Karlsruhe, Germany). The membranes were blocked with 5 % BSA solution for 1 h, and incubated for 4 h or overnight with rabbit monoclonal antibodies: GR, SOD1, GPx, Cat, GSS, phospho-p38^Thr180/Tyr182^, phospho-JNK^Thr183/Tyr185^, phospho-ERK1/2^Thr202/Thr204^, phospho-Akt^Ser473^, phospho-mTOR^Ser2448^, phospho-TSC2^Thr1462^, phospho-TSC2^Ser1254^, phospho-p70S6K^Thr389^, and PCNA or rabbit polyclonal antibodies: GCLC, and phospho-eIF4E^Ser209^ (all antibodies prepared 1:500 in 5 % w/v BSA or non-fat milk, 1× TBS, 0.1 % Tween-20). Alternatively, the membranes were blocked with 3 % non-fat milk-PBS solution for 2 h and incubated with rabbit nitrotyrosine polyclonal antibody overnight at 4 °C (2 μg/mL in 3 % w/v non-fat milk-PBS solution, 0.05 % Tween-20, pH = 7.4). Next, the membranes were incubated with a horseradish peroxidase-conjugated anti-rabbit antibody (1:5,000 for nitrotyrosine and 1:10,000 for the other antibodies in 1 × TBS) for 2 h. Chemiluminescence signals were detected on x-ray films. The membranes were stripped by re-blot buffer (10 mM Tris buffer saline, 10 % Tween-20, 200 mM NaOH and ddH_2_O), and incubated with rabbit monoclonal antibody against *â*-actin or rabbit polyclonal antibody against GAPDH as control.

### Statistical analysis

Data were expressed as mean ± standard error of the mean (SEM) of at least three rats per age group and subjected to One-Way Analysis of Variance (ANOVA) followed by Mann Whitney t-test (GraphPad Prism. 5 Software, San Diego, CA, USA). Values were considered to be statistically significant at values of P < 0.05.

## Results

### Liver damage and oxidative stress markers in serum and liver tissue

**Figure 1:**
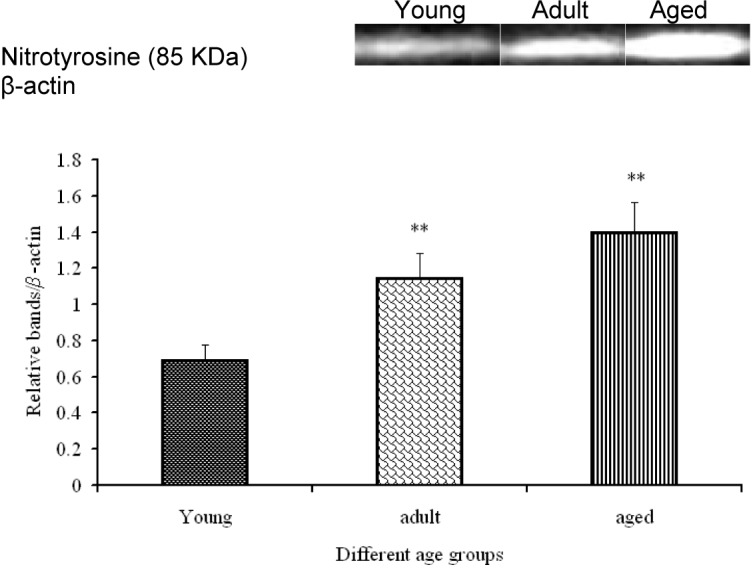
Nitrotyrosine protein levels in aging development of rat livers as determined by Western blot. Densitometric analysis of three rats per age group expressed as fold of β-actin depicted as mean ± SD. Significant differences (determined by One-way ANOVA) to the young group or between adult and aged rats are indicated (** p<0.01).

In Table 2(Tab. 2), several serum markers for liver damage and oxidative stress are listed. Serum ALT levels were significantly increased in the group of aged rats in comparison to those of young and adult rats. This finding indicates that the aged rats suffered from some degree of liver damage. Serum LDH values, however, were not altered statistically significantly. For the assessment of basal oxidative stress levels, 4-HNE was investigated. However, none of the tests has revealed significant differences among the three analyzed age groups (Table 2(Tab. 2)). Nitrotyrosine formation is accepted as a marker of cell and tissue damage as a result of oxidative stress (Choi et al., 2007[5]). We found that nitrotyrosine protein expression continuously increases with age, reaching its maximum in aged rat livers (Figure 1(Fig. 1)).

### Reduced and oxidized GSH in rat serum, liver cytosolic and mitochondria-enriched fractions

**Figure 2:**
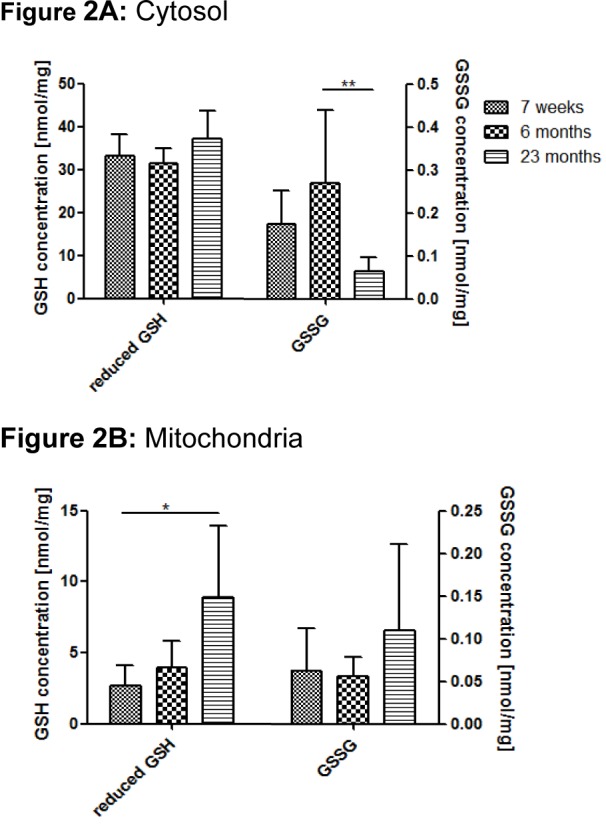
Reduced GSH and GSSG during aging rat liver cytosolic (A) and mitochondria-enriched (B) fractions as determined by photometric analysis. Values are mean ± SD of 6-8 rats per age group. Significant differences (determined by One-way ANOVA) to the young group (* p<0.05, ** p<0.01) are indicated.

Reduced and oxidized GSH have been measured in cytosolic and mitochondrial fractions, as well as in serum samples. Comparison of the three age groups revealed no statistical significance of cytosolic GSH during the development of aging. However, a slight peak of GSH was seen in old animals compared with the two younger groups (Figure 2A(Fig. 2)). On the other hand, the level of GSSG was higher in adult rats than in young animals. Interestingly, a significant reduction of GSSG was observed in aged rats (Figure 2A(Fig. 2)). In serum samples, the level of GSSG was higher in young and aged rats than in adult rats (Table 2(Tab. 2)). Mitochondria-enriched fractions have revealed a significant age-dependent increase of reduced GSH as well as of oxidized GSSG (Figure 2B(Fig. 2)).

### Expression of antioxidant and GSH-related genes in rat liver tissue

**Figure 3:**
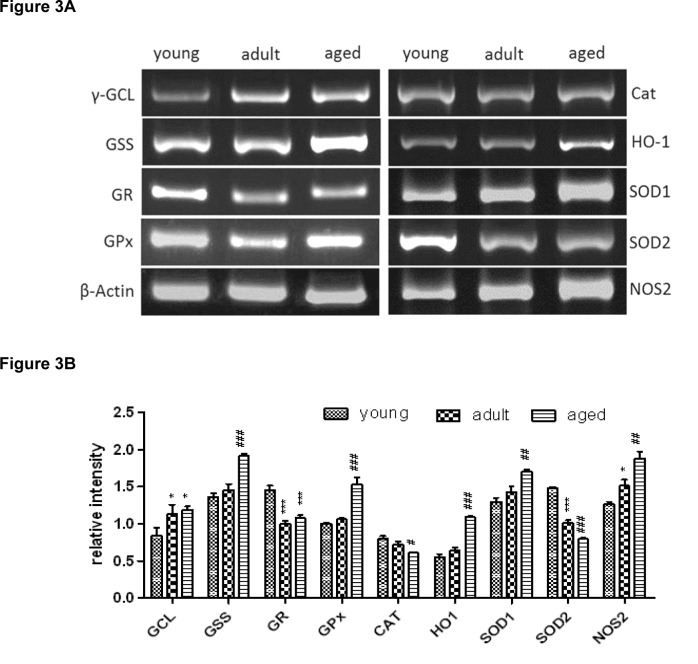
Gene expression of various antioxidant enzymes as determined by RT-PCR. (A) Original bands. (B) Densitometric analysis expressed as fold of β-actin is depicted as mean ± SD. Significant differences (determined by One-way ANOVA) to the young group (* p<0.5, *** p<0.01) or between adult and aged rats (# p<0.05, ## p<0.01, ### p<0.001) are indicated.

In Figure 3(Fig. 3), the expression of various antioxidant and GSH-related genes in rat liver tissue (expressed as fold of *β*-actin) as determined by RT-PCR is depicted. Genes involved in the *de novo* synthesis of GSH, i.e. γ-glutamylcysteine-ligase (γ-GCL) and glutathione synthetase (GSS), exhibit different trends. Regarding the rate-limiting enzyme γ-GCL, the gene expression levels remained almost constant during age development. However, GSS, which catalyzes the second step of the GSH synthesis, was significantly down-regulated in aged rats.

Compared to young rats, the gene expressions of GR and GPx, the enzymes for the recycling of GSH and GSSG, were slightly reduced in aged rats (Figure 3(Fig. 3)).

Moreover, different antioxidant enzymes were investigated at the gene expression level. The expression of CAT remained unchanged in all three age groups. SOD1 and the mitochondrial isoform SOD2 demonstrated a constant decrease in gene expression level during aging. A down-regulation in adult rats could be observed for heme oxygenase 1 (HO-1) and nitric oxide synthase 2 (NOS-2), however, in aged rats both genes were significantly up-regulated in comparison to adult rats.

### Antioxidant enzyme activities in rat liver tissue

**Figure 4:**
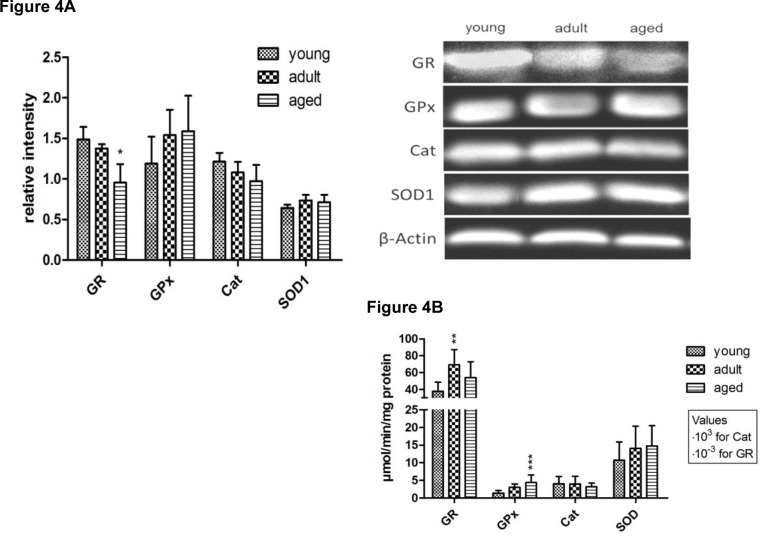
Selected antioxidant enzymes on protein (A) and enzyme activity level (B). Protein expression was determined by Western Blot (A, left) and analyzed densitometrically. Values are expressed as fold of β-Actin and are mean ± SD of three rats per age group (A, right). Enzyme activities (B) were determined by photometric or fluorometric activity assay and expressed as µmol/min/mg protein. At least 7 rats were analyzed per age group. Significant differences (determined by One-way ANOVA) to the young group (* p<0.05, ** p<0.1, *** p<0.01) are indicated.

In order to assess the antioxidant capacity of the livers of rats of different ages, selected enzymes were determined on protein level (Figure 4A(Fig. 4)), and liver cytosolic activities were measured (Figure 4B(Fig. 4)). SOD1 and CAT were determined as antioxidant enzymes. Furthermore, the GSH recycling capacity and the peroxide detoxification potential were assessed by GR and GPx activity measurement, respectively. Additionally, the protein expression levels of γ-GCL and GSS, the GSH-synthesizing enzymes were analyzed.

Although the protein level of SOD1 was significantly decreased in aged rats (Figure 4A(Fig. 4)), which confirms the gene expression data presented in Figure 3(Fig. 3), the cytosolic activity of SOD1 was increased during aging (Figure 4B(Fig. 4)). However, the induction on enzyme activity level was not statistically significant.

The cytosolic CAT activity demonstrated a slight age-dependent decrease at the protein and the enzyme activity level (Figure 4(Fig. 4)). The GSH recycling enzymes (GR and GPx) presented similar trends. In contrast to the gene expression of GPx and GR, which was reduced in aged rats (Figure 3(Fig. 3)), the protein as well as the catalytic activity of these enzymes increased significantly during aging (Figure 4A(Fig. 4) and B(Fig. 4)).

Furthermore, also the proteins of γ-GCL and GSS were significantly up-regulated during the aging process.

### Activation of MAPK involved in the regulation of mTOR

**Figure 5:**
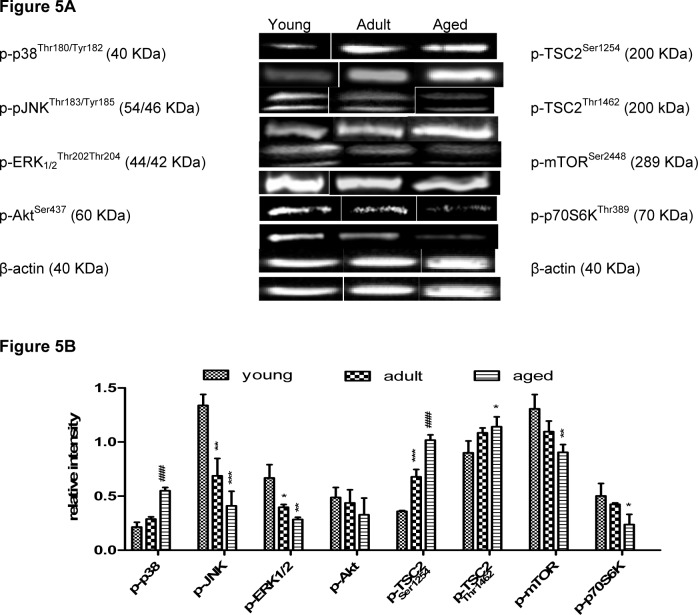
Phosphorylation status of selected MAPK and mTOR pathway components on protein level in rat liver tissue as determined by Western blot. (A) Original bands, (B) Densitometric analysis of three rats per age group expressed as fold of β-actin depicted as mean ± SD. Significant differences (determined by One-way ANOVA) to the young group (* p<0.5, ** p<0.1, *** p<0.01) or between adult and aged rats (# p<0.05) are indicated.

The phosphorylation of different MAPKs, which are known to be involved in the regulation of mTOR, was demonstrated by Western Blot analysis. Furthermore, the activation of up- and down-stream targets of the mTOR pathway, such as tuberous sclerosis complex 2 (TSC2) and p70S6K were analyzed. As depicted in Figure 5(Fig. 5), the phosphorylation of ERK1/2 increased significantly during aging, while the phosphorylation of JNK, p38, and Akt was significantly decreased in aged rats in comparison to adult ones. Regarding TSC2, one of the up-stream effectors of mTOR, two different phosphorylation sites, i.e. either TSC2^Ser1254^ (p-p38) and TSC2^Thr1462^ (p-Akt), were analyzed. Both sites exhibited a different trend of the phosphorylation during aging. On the one hand, the phosphorylation of TSC2^Thr1462^ increased significantly, and on the other hand, the p-TSC2^Ser1254^ slightly decreased in aged rats. As Akt activates mTOR via direct phosphorylation at Ser2448 (Youngman et al., 2011[55]), p-mTOR^Ser2448^ increased during aging (Figure 5(Fig. 5)). The phosphorylation of the mTOR down-stream target p70S6K, which regulates cell proliferation, was slightly increased in aged rats (Figure 5(Fig. 5)). 

### Regulation of proliferation

**Figure 6:**
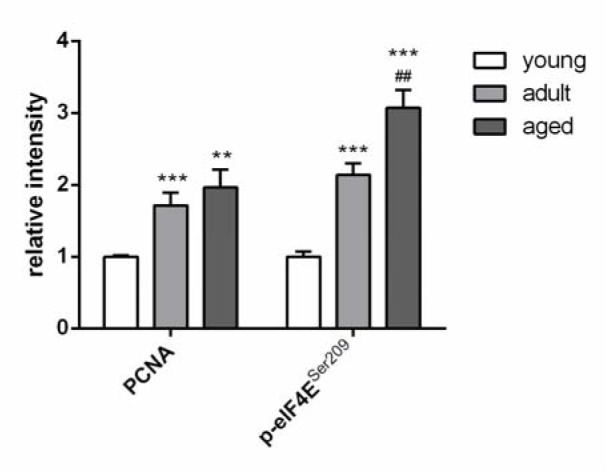
Protein expression of two different proliferation markers determined by Western Blot. Values are expressed as fold of GAPDH and are mean ± SEM of three rats per age group. Significant differences (determined by Mann Whitney t-test) to the young group (** p<0.01, *** p<0.001) or between adult and aged rats (^##^ p<0.01) are indicated.

Additionally, two other factors, which are involved in the regulation of the cell proliferation, were analyzed by Western Blot. The protein expression of proliferating cell nuclear antigen (PCNA) and the phosphorylation level of eukaryotic translation initiation factor 4E (eIF4E) significantly increased during aging (Figure 6(Fig. 6)).

## Discussion

It is widely accepted that the incidence of liver diseases increases during aging (Junaidi and Di Bisceglie, 2007[17], Grizzi et al., 2013[9]). However, in the current scientific discussion, age-related morphological and functional changes of the liver are of minor importance in comparison to those of other organic systems (Junaidi and Di Bisceglie, 2007[17]), although the death toll on the population which is older than 65 years is 3-5 times higher than on the population which is younger than 45 years. While the mechanisms of liver aging are not yet completely understood, oxidative stress has already been described as one of its possible contributors. Several reports have suggested that MAPK pathways are induced by ROS (Son et al., 2011[45], McCubrey et al., 2006[27]). Furthermore, MAPKs are known to directly or indirectly regulate the mTOR pathway, which integrates multiple signals from nutrients, growth factors, and oxygen to regulate cell growth, proliferation, and survival (Laplante and Sabatini, 2012[21]).

The aim of this study was the systematical determination of the age-dependent regulation of MAPK and mTOR signaling with regard to the (anti-)oxidant status in the liver of Wistar rats.

Altered activities of antioxidant enzymes may lead to an increase in oxidative stress, which, in turn, results in elevated levels of oxidative damage of cellular macromolecules and in a modified stress response (Lee et al., 2004[23]). Therefore, functional detoxification mechanisms for ROS and their reaction products, such as SOD and CAT, which directly detoxify ROS, are essential for cell survival (Inoue et al., 2003[15]). GPx protein expression and cytosolic GPx activity were significantly up-regulated in aged rats. Similar observations have been made by Zhu et al. (2006[60]) who have observed an increase in GPx activity in the brain of old rats as an adaptive effect to the age-related increase of peroxides. In our rat model, MDA in the serum of adult and aged rats was only slightly increased in comparison to the level in the serum of their young counterparts. This indicates a more pronounced lipid peroxidation in older animals. This, however, might not be a liver-specific phenomenon, as no increase of MDA was observed in the liver tissue samples. Due to the high activity of antioxidant enzymes, such as GPx and SOD, in aged animals, an even stronger lipid peroxidation in the liver might have been prevented. Reduced GSH is oxidized to GSSG by GPx during the catalytic cycle and recycling of GSSG by GR is important for the intracellular GSH homeostasis and GPx functionality (Ulusu and Tandogan, 2007[49]). We have observed that, in aged rats, the GR activity is higher than in young rats, but lower than in adult rats. Our observations were confirmed by several reports demonstrating a decrease in GR activity between 9 and 26 months-old rats, but also an increased GPx activity (Barja de Quiroga et al., 1990[2]).

As a consequence of the opposite age-dependent regulation of GPx and GR, one might expect a slow but successive increase in cellular GSSG concentration. However, such a shift in the GSH:GSSG ratio did not occur as had been expected: In aged rats, compared to young and adult Wistar rats, oxidized GSSG was significantly lower, while reduced GSH was higher. However, contrary to our results, Zhang et al. (2003[58]) have reported that the GSH:GSSG ratio is significantly lower in the livers of 24 months-old rats than in 6 months-old Fisher 344 rats. A study from Zhu et al. (2006[60]) has produced analogous results for the GSH:GSSG ratio in rat brains during aging. In contrast, it has also been reported that increased GSSG levels trigger the efflux of GSSG in the human liver and in human erythrocytes (Nur et al., 2011[32]). Therefore, we hypothesized that GSSG is actively transported into the blood stream and the bile. This hypothesis was confirmed later on by elevated serum GSSG levels in aged rats (Table 2(Tab. 2)). As a compensation, the amount of reduced GSH might be increased via the *de novo* synthesis pathway. On the protein expression level, both enzymes which are involved in GSH synthesis, γ-GCL and GSS, were significantly up-regulated during aging. Furthermore, the age-dependent increase of reduced GSH that was observed in rat liver cytosolic fractions underlines our hypothesis.

Serum ALT levels were significantly increased in aged rats. This indicates an increase of liver damage during aging. Similar results have been obtained by Ramesh et al. (2012[38]). As an indicator for lipid peroxidation, MDA was measured and it has been proven that only a slight age-dependent increase occurs in the serum, while no significant changes in tissue homogenates among the three investigated age groups have been found. However, the results from other studies on this topic are contradictory: Increased MDA values in livers of old rats have been reported (Zhang et al., 2003[58]; Mauriz et al., 2007[26]; Ramesh et al., 2012[38]) as well as no changes of MDA levels during aging (Tian et al., 1998[47]). These observations suggest that lipid peroxidation is not a suitable predictor of the aging process (Tian et al., 1998[47]).

In our study, direct ROS-detoxifying enzymes were only marginally affected by the aging process. Cytosolic CAT was only slightly decreased in aged Wistar rats on the protein expression, and on the activity level. Interestingly, however, data from the literature have not demonstrated any clear tendency on the effect of aging on the regulation of cytosolic CAT (Mauriz et al., 2007[26]). The age-related increase of the catalytic activity of SOD indicates a higher oxidative stress level in the aged rat livers. This confirms earlier data in the liver (Mauriz et al., 2007[26]), and in male rat erythrocytes (Ozturk and Gumuslu, 2004[34]). However, our studies also indicate that the gene and the protein expression of SOD1 are decreased during aging. The contrary results of protein amount and catalytic activity imply that the activity of SOD is regulated separately. A theory which explains these findings is the product inhibition of SOD by hydrogen peroxide (Gottfredsen et al., 2013[8]). As has been analyzed before, the GPx activity is also increased during aging, which leads to a reduction of the toxic hydrogen peroxide, and therefore the inhibition of SOD is abrogated. This reduced product inhibition, which has already been described earlier (Gottfredsen et al., 2013[8]), illustrates that a decreased SOD1 protein content can still lead to a higher enzyme activity. Some authors have also reported no change or even a decline in cellular SOD levels in different organs, including the liver (Santa Maria et al., 1996[42]). Possible explanations for these conflicting data are: analysis of different subcellular compartments, possible compensatory up-regulation or mitochondrial dysfunction caused by an age-dependent increase in cytosolic SOD activity. The latter is known to be a key factor in oxidative stress-mediated aging and related diseases (Cui et al., 2012[6]; Wang et al., 2014[50]).

Oxidative damage of mitochondria might be responsible for the ongoing degeneration, as mitochondrial DNA disposes of a high susceptibility to ROS. Therefore, a functional antioxidant system is essential for the maintenance of the integrity of mitochondria. It is believed that SOD2 plays a pivotal role in this maintenance (Okado-Matsumoto and Fridovich, 2001[33]). Similar to cytosolic SOD1, mitochondrial SOD2 was also significantly decreased on the gene expression level in the aging rat liver. However, the protein expression of SOD2 is constantly and significantly increased during the aging process. SOD2 activity and mitochondrial DNA damage were increased in the livers of human subjects during aging (Yen et al., 1994[53]). Furthermore, in male Fischer 344 rats, a clear age-dependent increase of SOD2, together with elevated mitochondrial GPx activity and a decline of GR activity was observed (Rikans et al., 1992[40]).

In our study, the level of reduced GSH in the mitochondria significantly increased during aging. This observation indicates the necessity of an increased demand of defense against oxidants in the mitochondria of aged Wistar rats. It is known that the GSH *de novo* synthesis exclusively takes place in the cytosol, and that, therefore, mitochondrial GSH is regulated by an active transport which is carried out by an organic anion transporter, such as oxoglutarate and dicarboxylate carrier (Lash, 2006[22]). GSSG also increased from young to aged rats. This observation indicates a rise of oxidative stress in the mitochondria with increasing age. Besides the antioxidant status of the rat liver, we have determined the activation of Akt, MAPK, and several components of the mTOR pathway by detecting the phosphorylation status via Western Blot analysis. Besides the activation of mTOR itself and the down-stream target p70S6K, two different phosphorylation sites on the up-stream complex TSC1/2 were analyzed. The phosphorylation of Thr1462 by Akt results in the inactivation of TSC2 by the formation of a complex with TSC1, thereby the GAP activity of TSC2 is blocked and the GTPase activity of Rheb is inhibited. The GTP-bound Rheb is a direct activator of mTORC1 (Jham et al., 2011[16]). As a consequence, the phosphorylation of the down-stream targets of mTOR, such as p70S6K, 4E-BP1 and RS6P, are activated. These activations influence crucial cellular processes, such as proliferation, apoptosis, autophagy, and, finally, cell survival (Laplante and Sabatini, 2012[21]; Weichhart and Saemann, 2009[51]). In contrast, phosphorylated Ser1254 leads to the activation of TSC2. Huang and Manning (2008[14]) have demonstrated that p38 phosphorylates TSC2S^er1254^ through mitogen-activated protein kinase kinase 2 (MKK2), and therefore inhibits the mTOR complex.

Our data have demonstrated that the phosphorylation level of Akt^Ser473^ increases only insignificantly from young to adult rats. Although p-Akt^Ser473^ decreases from adult to aged rats, p-TSC2^Thr1462^ constantly and significantly increases during aging. These discrepancies of the results can be explained by the fact that p-TSC2^Thr1462^ is also a target of other kinases, like *e.g.* ERK1/2 (Roux et al., 2004[41]). At the same time, p-p38^Thr180//Tyr182^ and, in turn, p-TSC2^Ser1254^ are slightly reduced in aged rats. These results imply that the inhibition of the TSC1/2 complex is amplified in the aged rat liver. Therefore, the activation of mTOR is increased during aging, which fits to our results and this, in turn, leads to an increased proliferation rate. This result is in accordance with the observed slight increase of the phosphorylated down-stream target p70S6K which regulates cell proliferation. Additionally, PCNA, which regulates the DNA replication, and p-eIF4E^Ser209^, which controls the initiation of translation, were analyzed (Palaniappan and Menon, 2010[35]). Both proliferation markers have demonstrated an elevated protein level in aged rats which proves the increased cell proliferation rate in the liver of rats. This analysis matches the theory by Nelsen et al. (2001[31]) that liver cells only proliferate in cases of necessity, like *e.g.* injury and cell damage, to maintain liver function. Furthermore, p-mTOR^Ser2448^ is directly phosphorylated by Akt, which leads to a doubled effect on the activation of cell proliferation (Zeng et al., 2007[57]). Recent studies have confirmed our results, showing that p-Akt^Ser473^ is able to activate mTOR in hematopoietic stem cell aging by phosphorylation of Ser2448, leading to an increase of down-stream p-p70S6K^Thr389^ (Chen et al., 2009[4]).

Aging tissues develop characteristics of chronic stress, such as lipid peroxidation and ROS formation (Hsieh et al., 2003[13]). In our rat model, we have detected only moderate signs of liver damage and increased oxidative stress in the aging rat liver. At the signaling level, we would have expected that stress-related pathways might change with increasing age. Therefore, we analyzed the phosphorylation levels of p38^Thr180//Tyr182^, JNK^Thr183/Tyr185^ and ERK1/2^Thr202/Thr204^, and have been able to demonstrate that the phosphorylation of ERK1/2 is significantly increased in the aging rat liver. Roux et al. (2004[41]) have demonstrated that, similar to p-Akt, p-ERK1/2 also phosphorylates p-TSC2^Thr1462^ among others. Thus, the activation of ERK1/2 also leads to a phosphorylation of mTOR and thereby to an increase in cell proliferation. Previous publications have demonstrated that p-ERK1/2 regulates positively the Nrf2 pathway, which, in turn, controls the glutathione homeostasis among others. For example, γ-GCL is a target of Nrf2. Thus, the activation of ERK1/2 is linked to the up-regulation of the *de novo* synthesis of glutathione (Tufekci et al., 2011[48]). Our observation of an increased protein expression of *γ*-GCL in aged rats supports this hypothesis.

Nitrotyrosine, a marker for oxidative damage, has been detected in old cells and in damaged tissue (Choi et al., 2007[5]; Yin et al., 2009[54]). Our data exhibit an increased formation of nitrotyrosine from young to adult and reaching its maximum in aged rat livers. Similar trends have been observed for p-Akt^Thr1462^, p-p38^Thr180//Tyr182^, and p-JNK^Thr183/Try185^ in the aging liver. This finding is in agreement with data showing that nitrotyrosine (oxidative stress) appears to be in correlation with the generation of ROS as well as the activation of MAPKs (Mu et al., 2008[29]).

## Conclusion

Altogether, we have demonstrated that several changes in the antioxidative system and in stress-related signaling pathways in the livers of Wistar rats occur during aging. The activation of the mTOR pathway seems to be stimulated in aged rat livers, and hence the cell proliferation capacity, which is measured by the phosphorylation of down-stream mTOR-p70S6K, as well as by the protein contents of PCNA and p-eIF4E^Ser209^, is increased in aging animals. This fact is a possible explanation for the increased regeneration capacity in situations of increased damage (e.g.; liver resections, hepatitis) or in the aged liver. The impact of aging on the mTOR signaling in the liver has to be further investigated, as not only proliferation but also other important processes, such as apoptosis or autophagy are controlled by mTOR.

## Notes

Wei Yang, Britta Burkhardt and Luise Fischer contributed equally as first authors.

## Conflict of interest

The authors declare that there is no conflict of interests regarding the publication of this paper.

## Acknowledgements

The work for this study has been partially funded by the Federal Ministry of Education and Research (BMBF, FKZ 0315891A, 0315891C, 0315891D).

## Figures and Tables

**Table 1:**
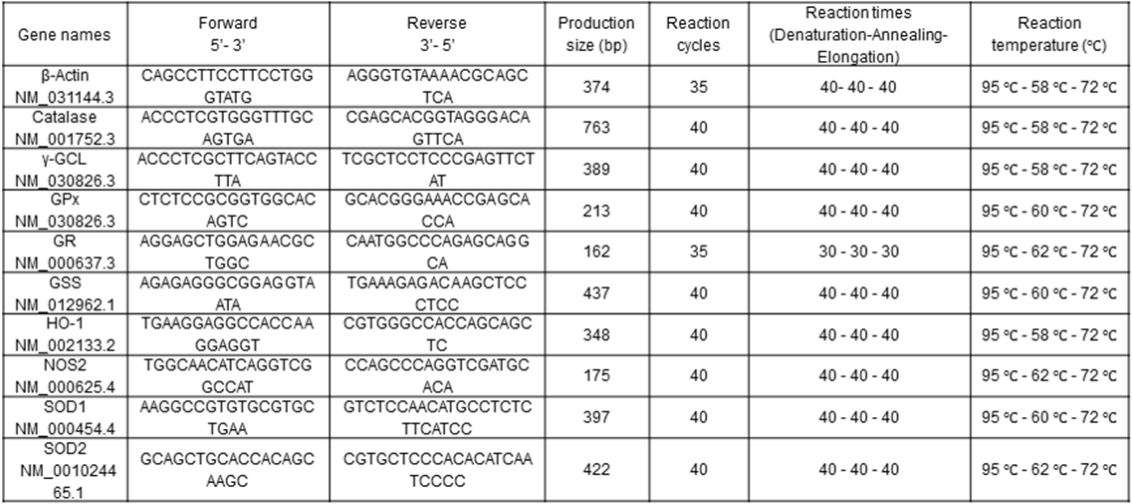
Primer pairs for antioxidase and *β*-actin

**Table 2:**
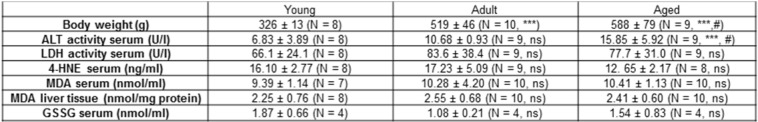
Serum markers of liver and tissue damage (ALT, LDH), lipid peroxidation marker 4-HNE in serum, and serum concentrations of GSSG of young (7 weeks), adult (6 - 7 months), and aged (23 months) rats
